# Supplementary material for: Relationships between the hard and soft dimensions of the nose in Pan troglodytes and Homo sapiens reveal the positions of the nasal tips of Plio-Pleistocene hominids
Source: PLoS One. 2022 Feb 22;17(2):e0259329. doi: 10.1371/journal.pone.0259329 (PMC8863275; doi:10.1371/journal.pone.0259329)
Supplement: S1 Table — (DOCX) [file pone.0259329.s001.docx]

**Supplementary Table 1**

List of non-human primate subjects included in this study.

| **Cat No.** | **ID** | **Species** | **Specimen condition** | **Sex** | **Age** | **Source** |
| --- | --- | --- | --- | --- | --- | --- |
| 366 | PRI-Cleo | *Pan troglodytes* | living | F | 9 | KUPRI |
| 367 | PRI-Pal | *Pan troglodytes* | living | F | 10 | KUPRI |
| 365 | PRI-Popo | *Pan troglodytes* | living | F | 27 | KUPRI |
| 364 | PRI-Pen | *Pan troglodytes* | living | F | 33 | KUPRI |
| 274 | PSI-Akira | *Pan troglodytes* | living | M | 34 | KUPRI |
| 467 | PRI-Mari | *Pan troglodytes* | living | F | 35 | KUPRI |
| 456 | PRI-Reiko | *Pan troglodytes* | living | F | 44 | KUPRI |
| 690 | TZ-Apple | *Pan troglodytes* | fresh | F | 30 | KUPRI |
| 659 | PRI-9803 | *Pan troglodytes* | fresh | M | 34 | KUPRI |
| 1523 | HCZ-Chieko | *Pan troglodytes* | fresh | F | 40 | KUPRI |
| 1486 | PRI-10814 | *Pan troglodytes* | fresh | F | 43 | KUPRI |
| 573 | PRI-9783 | *Pan troglodytes* | fresh | F | 44 | KUPRI |
| 26 | KCZ-Yoko | *Pan troglodytes* | frozen | F | 20 | KUPRI |
| 318 | PRI-9262 | *Pan troglodytes* | frozen | M | 22 | KUPRI |
| 1320 | PRI-10301 | *Pan troglodytes* | frozen | M | 24 | KUPRI |
| 455 | PRI-9457 | *Pan troglodytes* | frozen | M | 32 | KUPRI |
| 788 | HAZ-Yuko | *Pan troglodytes* | frozen | F | 42 | KUPRI |
| 344 | PRI-9266 | *Pan troglodytes* | immersed | M | 29 | KUPRI |
| 485 | PRI-9473 | *Pan troglodytes* | immersed | M | 35 | KUPRI |
| 650 | PRI-7895 | *Pan troglodytes* | immersed | - | approx. 3 | KURPI |
| M201606 | S12652 | *Pan troglodytes* | preserved | F | 5 | Morphosource |
| - | - | *Homo sapiens* | living | M | 29 | Donated |
| M20189 | S9655 | *Pan paniscus* | wet specimen | M | 4 | Morphosource |
| 269 | PRI-7902 | *Gorilla gorilla* | immersed | - | approx. 3 | KUPRI |
| 296 | PRI-Oki | *Gorilla gorilla* | fresh | F | 54 | KUPRI |
| 317 | PRI-Willie | *Gorilla gorilla* | fresh | M | 46 | KUPRI |
| 736 | PRI-Satsuki | *Pongo pygmaeus* | frozen | F | 42 | KUPRI |
| _ | Puspa | *Pongo abelii* | living | F | 46 | Zoos SA |
| 329 | PRI-9264 | *Symphalangus syndactylus* | frozen | F | 1 | KUPRI |
| 753 | PRI-Setsu | *Symphalangus syndactylus* | frozen | M | 8 | KUPRI |
| 1011 | PRI-10050 | *Symphalangus syndactylus* | fresh | M | - | KUPRI |
| 1154 | PRI-7719 | *Papio hamadryas* | immersed | F | - | KUPRI |
| 1025 | PRI-2868 | *Papio hamadryas* | immersed | F | - | KUPRI |
| 241 | PRI-Ph83 | *Papio hamadryas* | living | M | 17 | KUPRI |
| - | Sts 5 | *Australopithecus africanus* | fossil | - | - | Wits University |
| - | MH1 | *Australopithecus sediba* | fossil | - | - | Morphosource |
| - | KNM-WT 17000 | *Paranthopus aethiopicus* | fossil | - | - | HETMP |
| - | OH5 | *Paranthropus boisei* | fossil | - | - | Morphosource |
| - | KNM-ER 1813 | *Homo habilis* | fossil | - | - | Morphosource |
| - | KNM-WT 15000 | *Homo ergaster / erectus* | fossil | - | - | African Fossils |
| - | LES1 | *Homo naledi* | fossil | - | - | Morphosource |
| - | Kabwe 1 | *Homo rhodesiensis / heidelbergensis* | fossil | - | - | Morphosource |
| - | Amud 1 | *Homo neanderthalensis / Neandertals* | fossil | - | - | Morphosource |
