# Supplementary material for: Relationships between the hard and soft dimensions of the nose in Pan troglodytes and Homo sapiens reveal the positions of the nasal tips of Plio-Pleistocene hominids
Source: PLoS One. 2022 Feb 22;17(2):e0259329. doi: 10.1371/journal.pone.0259329 (PMC8863275; doi:10.1371/journal.pone.0259329)
Supplement: Attachment — Submitted filename: Ethics Waiver.pdf [file pone.0259329.s003.pdf]

THE UNIVERSITY of ADELAIDE

RESEARCH SERVICES
OFFICE OF RESEARCH ETHICS, COMPLIANCE AND INTEGRITY
THE UNIVERSITY OF ADELAIDE

LEVEL 3, RUNDLE MALL PLAZA
50 RUNDLE MALL
ADELAIDE SA 5005 AUSTRALIA

TELEPHONE +61 8 8313 6028
FACSIMILE +61 8 8313 3700
EMAIL hrec@adelaide.edu.au

CRICOS Provider Number 00123M

24 January 2022

Mr Ryan M. Campbell
School of Biomedicine
Faculty of Health and Medical Sciences
The University of Adelaide

Dear Mr Campbell,

Reconstructing Soft Tissues of Plio-Pleistocene Hominids using Comparative Anatomy of Modern humans and Great apes

Thank you for your email dated 12 January 2022 and subsequent correspondence detailing the above project, supervised by Dr Jaliya Kumaratilake, Professor Maciej Henneberg, and Dr Fernando Marmolejo-Ramos.

Whilst the University Human Research Ethics Committee does not provide retrospective review and approval, or exemption in this case, the Human Research Ethics Secretariat (HRES) has considered the information provided and can confirm that the research conducted in this project would have been suitable for an exemption.

As your project carried negligible risk and involved the use of non-identifiable, pre-existing data about human beings, it met the requirements of section 5.1.22 in the *National Statement on Ethical Conduct in Human Research 2007 (Updated 2018)* regarding research that can be exempted from review and therefore would not have required formal ethical review by the University of Adelaide Human Research Ethics Committee.

Yours sincerely,

Dr Monica Kerr
Manager, Office of Research Ethics, Compliance, and Integrity
