# Supplementary material for: Relationships between the hard and soft dimensions of the nose in Pan troglodytes and Homo sapiens reveal the positions of the nasal tips of Plio-Pleistocene hominids
Source: PLoS One. 2022 Feb 22;17(2):e0259329. doi: 10.1371/journal.pone.0259329 (PMC8863275; doi:10.1371/journal.pone.0259329)
Supplement: Attachment — Submitted filename: Plos ONE Dimensions of the nose.docx [file pone.0259329.s002.docx]

**Plos ONE Dimensions of the nose**

Thank you for the opportunity to review this paper. I think this is a very interesting idea, as little information is indeed available on the relationship between hard and soft tissues in non-human primates. In fact, the large body of research that deals with this topic in human subjects attests to the complexity of this issue. This said, I have a few concerns:

1. The authors use quite a simple method, based on a few linear measurements (through regression analysis) and angles of the skull, to predict the position of the tip of the nose (pronasale). They refer to the basion-prosthion / basion-pronasale measurements as nasal cavity lengths, while in fact this measurement reflects much more than just the nasal cavity – it also encompasses aspects of the cranial base that are overlooked. While the similarity between the great apes and humans are interesting for these regressions, the results are only usable to very roughly predict the position of the tip of the nose. This should be acknowledged. On the second page of the Introduction, the authors list a few methods /references that have been used (in humans) to establish pronasale - this list is quite outdated and does not really illustrate the complexity of the problem. In this regard, see Carl N. Stephan, Jodi M. Caple, Pierre Guyomarc’h & Peter Claes (2019) An overview of the latest developments in facial imaging, Forensic Sciences Research, 4:1, 10-28, references 66 – 80 for a more recent view on this issue.
2. The sample sizes used in this study are quite small, as acknowledged by the authors. This in itself should encourage the authors to be more cautious in their discussion as to the usability of these formulae. While one can understand the paucity of primate data, I found the human reference sample problematic. It included only Chinese and American/European individuals and does not include any African samples. This should be corrected, as a sample of individuals who tend to be more prognathic would most certainly have strengthened the results and added more variation. I do not think the current sample adequately represents human variation.
3. In the Discussion, the authors talk at length about possible reasons (or not) for the development of protruding noses in humans. This goes beyond the scope of this paper and also beyond what can be said from the available data. It is also not reflected in the title of the paper. This section should be shortened, to only get to the crux of the matter, namely that these formulae may be helpful to very approximately estimate the position of pronasale (with a SE of 5 mm, which is quite considerable in actual terms – it is often helpful to assess this in terms of the percentage difference that this would make in an actually reconstructed feature).
4. I wondered about the issue of no ethics permission needed. The reference data (lateral cephalometric radiographs) being of humans would require some sort of permission. In our institution, an ethics waiver exists for data that are curated for research purposes. A committee oversees the granting this waiver to researchers, making sure that the data are used for ethical purposes. Some similar sort of permission from the gatekeepers of this database should be included.

Other issues:

1. Intro, 2^nd^ para: the statement “It has been stated that primate comparative anatomy..” needs a reference.
2. The aim statement needs clarification – “The aims of this study were to explore this matter further” – which matter? Clearly state the aim. Also in the aim it is stated that the aim will be achieved “secondly by performing a set of out-of-group test” – of what (who are the out of group individuals) and why?
3. I am concerned about the inclusion of juveniles (p. 16). This has not been properly motivated and adds unnecessary complexity. This should be better motivated and discussed or else left out.
4. The TEM assessments – were these inter- or intra-observer repeat assessments?
5. P. 9, bottom, the relationship between nasal size and cranial size examined – not really cranial size, which is much more than the few dimensions used here.
6. P. 10 2^nd^ para: To DEMONSTRATE the reliability…. Change demonstrate to assess, as we do not want to pre-empt the results (demonstrate), but rather want to assess whether there is a relationship.
7. Training sample – two subjects only. This is really quite s small dataset which limits the results of the study.
8. P. 11 – it is nice that the method was tested on hominids, but of course this is only a nicety in this context and does not really prove anything. It shows if realistic-looking reconstructions are possible using this method, and nothing more. This should be clearly stated.
9. In the first part of the Results section, the p-values are stated. The manner in which this is stated should be relooked – for example it is stated that “Average nasal cavity length of chimpanzees is SOMEWHAT GREATER than that of modern humans…”, but the p value is quoted as p<0.001. This is highly statistically significant. Also the “Average na-ba-pr angle of chimpanzees is ONLY SLIGHTLY smaller than that of modern humans” but again the p-value is <0.001. Are you trying to convince us of your point?
10. In the next paragraph – “The rations (ratios) were rather similar….” – they are not really similar 122.1 versus 115.8. Also the other ratios quoted. These need to be put into perspective.
11. Discussion: as said before, the discussion overinterprets the available results and should be shortened to include only what can be realistically said from these results.
